# The Role of Antioxidation and Immunomodulation in Postnatal Multipotent Stem Cell-Mediated Cardiac Repair

**DOI:** 10.3390/ijms140816258

**Published:** 2013-08-06

**Authors:** Arman Saparov, Chien-Wen Chen, Sarah A. Beckman, Yadong Wang, Johnny Huard

**Affiliations:** 1 Nazarbayev University Research and Innovation System, Nazarbayev University, Astana 010000, Kazakhstan; 2 Department of Orthopedic Surgery, University of Pittsburgh, Pittsburgh, PA 15219, USA; E-Mails: chc88@pitt.edu (C.-W.C.); beckman.sarah@gmail.com (S.A.B.); 3 Department of Bioengineering, University of Pittsburgh, Pittsburgh, PA 15261, USA; E-Mail: yaw20@pitt.edu; 4 Stem Cell Research Center, University of Pittsburgh, Pittsburgh, PA 15219, USA; 5 Department of Molecular Cardiovascular Biology, Cincinnati Children’s Hospital Medical Center, Cincinnati, OH 45229, USA; E-Mail: beckman.sarah@gmail.com; 6 McGowan Institute for Regenerative Medicine, University of Pittsburgh, Pittsburgh, PA 15219, USA

**Keywords:** stem cells, myocardial infarction, oxidative stress, inflammation, immune system, cardiac repair

## Abstract

Oxidative stress and inflammation play major roles in the pathogenesis of coronary heart disease including myocardial infarction (MI). The pathological progression following MI is very complex and involves a number of cell populations including cells localized within the heart, as well as cells recruited from the circulation and other tissues that participate in inflammatory and reparative processes. These cells, with their secretory factors, have pleiotropic effects that depend on the stage of inflammation and regeneration. Excessive inflammation leads to enlargement of the infarction site, pathological remodeling and eventually, heart dysfunction. Stem cell therapy represents a unique and innovative approach to ameliorate oxidative stress and inflammation caused by ischemic heart disease. Consequently, it is crucial to understand the crosstalk between stem cells and other cells involved in post-MI cardiac tissue repair, especially immune cells, in order to harness the beneficial effects of the immune response following MI and further improve stem cell-mediated cardiac regeneration. This paper reviews the recent findings on the role of antioxidation and immunomodulation in postnatal multipotent stem cell-mediated cardiac repair following ischemic heart disease, particularly acute MI and focuses specifically on mesenchymal, muscle and blood-vessel-derived stem cells due to their antioxidant and immunomodulatory properties.

## 1. Introduction

Cardiovascular disease is the leading cause of mortality in the world with over 17 million deaths in 2008 alone [1], which is estimated to increase to over 23 million by 2030 and to remain the leading cause of deaths [2]. Coronary heart disease causes the majority of deaths in cardiovascular disease with myocardial infarction (MI) often leading to the development of heart failure. MI is caused by occlusion of a coronary artery, which leads to a deficiency of oxygen and nutrients at the site of infarction and the subsequent death of cardiomyocytes. The exposure of danger-associated molecular patterns (DAMPs) from dead cardiomyocytes, triggers the migration of neutrophils, monocytes, dendritic cells, lymphocytes and other cells with subsequent initiation of inflammation, oxidative stress, tissue regeneration and cardiac remodeling; however, the mechanisms behind these processes are still not fully understood [3–6].

Stem cell therapy is considered a promising approach in treating coronary heart disease including MI due to the ability of stem cells and/or their secretory factors to influence host cell migration, cellular functions, cardiomyocyte survival, tissue regeneration and healing [7–9]. The use of stem cells and/or their secretory factors may lead to effective therapeutic approaches for reducing the infarct size, generating more functional heart tissue, eliminating heart failure and improving the life of patients who have suffered from MI. The current review highlights the role of oxidative stress and the immune system in pathogenesis of acute MI and focuses specifically on mesodermal stem cells, including mesenchymal, muscle and blood-vessel-derived stem cells, due to their antioxidant and immunomodulatory properties.

## 2. Oxidative Stress and Inflammation Following Myocardial Infarction

Oxidative stress and inflammation play major roles in tissue damage, clearance of cell debris, myocardial fibrosis, and remodeling of heart tissue following MI, although the details of the initiation, development and control of these processes have not been fully deciphered. In this section, we briefly review the sequence of events related to oxidative stress and inflammation mediated by the cells of the immune system to better understand possible therapeutic targets using stem cells or their factors described in the following sections.

**Figure 1:**
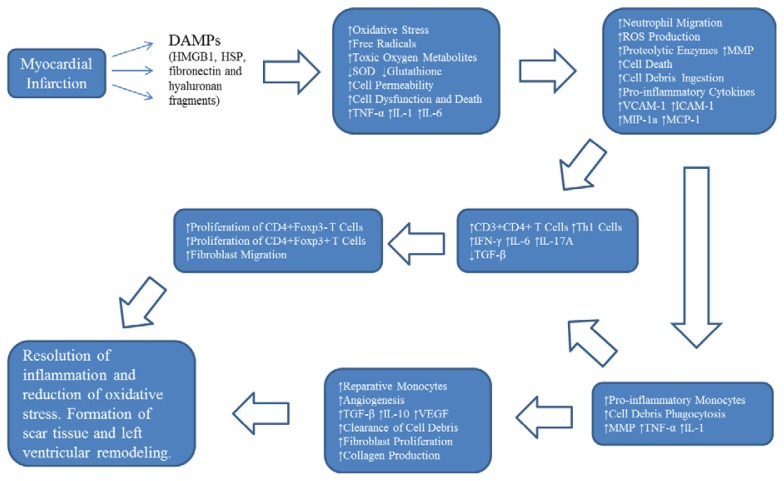
Schematic depiction of representative events following myocardial infarction (MI). MI causes the exposure of danger-associated molecular patterns (DAMPs), initiation of oxidative stress and inflammation, and can lead to the resolution of inflammation, reduction of oxidative stress, formation of scar tissue and left ventricular remodeling. Nevertheless, the pathological processes following MI are very complex with overlaps of depicted events and participation of additional cell types/secretory factors that are not included in this figure.

MI causes necrosis and apoptosis of cardiomyocytes and exposure of DAMPs, which are molecules produced by stressed/damaged cells or extracellular matrix fragments, and initiate the innate immune response and inflammation [10]. The DAMPs that are involved in MI are mainly high motility group box 1 (HMGB1), heat shock proteins (HSPs), fibronectin and hyaluronan fragments (Figure 1). HMGB1, a chromatin binding protein located in the nucleus, which can be secreted from the stressed cells and form a heterodimer with IL-1β [11], stimulates the production of pro-inflammatory cytokines such as TNF-α, IL-1 and IL-6 by macrophages after binding to Toll-like receptor (TLR) 4 [12,13]. In addition, HMGB1 enhances cell migration by forming a heterocomplex with chemokine CXCL12, which is mediated by CXCR4, the receptor for CXCL12 [14]. HSP60, which is a stress protein released from cardiac myocytes after hypoxic shock, can induce the expression of pro-inflammatory cytokines TNF-α and IL-1β, activate caspase 3 and 8, as well as cause DNA fragmentation and apoptosis of cardiac myocytes by binding to TLR4 [15,16]. Both fibronectin and hyaluronan fragments, which are both constituents of the extracellular matrix, can activate the innate immune system by binding to TLR2 and TLR4 [17,18].

DAMPs are recognized by TLR on the surface of cardiac myocytes, endothelial cells and cells of the immune system. TLR in humans were discovered by Madzhidov and colleagues [19] and are pattern recognition receptors and a part of the innate immune system. TLR4 plays a key role in the response of the innate immune system following MI. Indeed, the TLR4 knockout animals demonstrated diminished inflammation, a reduction in the number of apoptotic cells and CD3+ T cells in the infarcted area, attenuated left ventricular remodeling and improved animal survival [20,21]. Similarly, the TLR2 knockout mice showed better survival, reduced fibrosis and downregulation of TGF-β expression in the heart tissue [22]. TLR binding results in the recruitment of myeloid differentiation primary response 88 (MyD88), and eventually, the translocation of NF-κB into the nucleus and activation of chemokine and pro-inflammatory cytokine gene expression [23,24]. Furthermore, components of the apoptotic cardiac myocytes and DAMPs can activate the complement system that plays an active role in the pathogenesis of MI [25,26]. Cardiac myocytes express the receptor to C5a, which is up-regulated following MI, and antibodies against C5a decrease infarct size, reduce vascular permeability and adherence of neutrophils to endothelial cells in experimental models of MI [27,28].

Oxidative stress, which results from the excessive production of reactive oxygen species (ROS) and the inability of the antioxidant system to neutralize them, plays a pivotal role in damaging and remodeling cardiac tissue. ROS are oxygen-containing compounds such as superoxide anion, hydroxyl radical and hydrogen peroxide, mainly produced in the heart by neutrophils, cardiac myocytes and endothelial cells. Myocardial ischemia leads to increased ROS activity through two mechanisms: (1) a decrease in the defensive activity including reduced mitochondrial superoxide dismutase function and diminished reserve of reduced glutathione, and (2) an increase in the offensive action including elevated production of free radicals and toxic oxygen metabolites, especially following the post-ischemic reperfusion [29]. ROS cause cell apoptosis by breaking down DNA and stimulating pro-apoptotic signaling pathways. In addition, ROS induce the production of pro-inflammatory cytokines and matrix metalloproteases (MMP) that further aggravate inflammation and pathological remodeling. The regulation of gene expression by ROS is mediated by NF-κB, AP-1 and peroxisome proliferator-activated receptor. Oxidative stress causes endothelial cell dysfunction, up-regulation of adhesion molecules, secretion of pro-inflammatory cytokines, and increase of endothelial cell permeability and migration of neutrophils [30,31].

Neutrophils are the first cells of the immune system that migrate to the site of injury in the heart and produce ROS and proteolytic enzymes (Figure 1). The migration of neutrophils is mediated by up-regulated P-selectin (CD62P) and E-selectin (CD62E) on the surface of endothelial cells [32] and up-regulated L-selectin (CD62L) on the surface of neutrophils [33] as well as an enhanced ability of intercellular cell adhesion molecule-1 (ICAM-1) on the surface of endothelial cells to bind neutrophils [34]. Double knockout mice for P-selectin and ICAM-1 demonstrated a significantly reduced migration of neutrophils without affecting the infarct size [35]. C5, a factor of activated complement, and IL-8 play important roles in neutrophil chemotaxis [36]. The recruited neutrophils ingest dead cell debris, produce MMP to degrade the extracellular matrix, release ROS, and secrete proteolytic enzymes and pro-inflammatory cytokines that further aggravate inflammation. Neutrophils can bind cardiac myocytes via ICAM-1 and directly damage them by producing ROS and proteolytic enzymes [37]. In addition, neutrophils contribute to the initiation of monocyte migration by up-regulating adhesion molecules and by producing chemotactic factors. Neutrophils secrete granules that up-regulate the expression of vascular cell adhesion molecule-1 (VCAM-1) and ICAM-1 on the surface of endothelial cells, stimulate production of IL-8 and macrophage inhibitory protein-1a (MIP-1a) by monocytes and macrophages, and monocyte chemotactic protein-1 (MCP-1) by endothelial cells [38].

Nahrendorf and colleagues demonstrated the biphasic migration of monocytes to the heart tissue in a mouse model of MI [39]. The first population of monocytes that possess pro-inflammatory properties migrate to the site of inflammation via a MCP-1 dependent pathway during the first four days after infarction, phagocytose cell debris, produce MMP which degrade the extracellular matrix and express pro-inflammatory cytokines TNF-α and IL-1β (Figure 1). The second population of monocytes with reparative properties stimulates angiogenesis, expresses TGF-β, IL-10 as well as vascular endothelial growth factor (VEGF), and can be found at the site of inflammation starting by day four after MI with the recruitment mediated by fractalkine. The turnover of monocytes in the infarcted heart is about twenty hours with the death of the majority of cells at the site of inflammation and only a minority of undifferentiated monocytes remaining which can be found mainly in the blood and liver, with smaller numbers present in the spleen and lymph nodes. The spleen serves as a main source of newly differentiated monocytes, and IL-1β plays an essential role in monocytopoesis [40]. Differentiated macrophages phagocytose dead cells and cell debris and clear apoptotic neutrophils and cardiac myocytes. Apoptotic neutrophils produce factors that inhibit neutrophil recruitment and contribute to the production of TGF-β and IL-10 by macrophages [41]. On the other hand, macrophages produce cytokines that stimulate fibroblast proliferation, angiogenesis and the production of collagen. The differentiated macrophages can be divided into two major subsets: (1) classically activated M1, which are stimulated by IFN-γ, and (2) alternatively activated M2, which are induced by IL-4 and IL-13 [42,43]. In addition to neutrophils and monocytes/macrophages, dendritic cells (DC) also play an important role in the pathogenesis of MI. Anzai and colleagues reported that DC migrate to the site of injury reaching their peak concentration at one week following MI and the deletion of DC adversely affected left ventricular remodeling and cardiac function. Furthermore, the deletion of DC caused the elevation in the expression of pro-inflammatory cytokines and a reduction in the level of anti-inflammatory cytokines as well as a shift towards the accumulation of pro-inflammatory monocytes/macrophages in the infarcted myocardium [44].

T lymphocytes are instrumental in the regulation of the immune response and the response following myocardial infarction is no exception. There are two major subtypes of T cells: CD4+ and CD8+ lymphocytes. CD4+ T cells can be divided into several populations based on their functional activity and cytokine profile: Th1 cells produce IFN-γ and are responsible for the defense against intracellular pathogens, Th2 cells secrete IL-4, IL-5 and IL-13 and play a pivotal role in the clearance of extracellular parasites, Th17 cells are characterized by IL-17 production and participate in inflammation and autoimmunity, and regulatory T cells secrete TGF-β and IL-10 to regulate the immune response [45–47]. In contrast, CD8+ T lymphocytes are cytotoxic cells mainly responsible for killing infected and tumor cells [48]. Maisel and colleagues demonstrated that the adoptive transfer of *ex vivo* activated splenocytes, isolated from animals with MI, into healthy syngeneic animals’ caused myocardial injury with predominantly lymphocyte and plasma cell infiltration. The injury was cardiac specific with a good correlation between the infarct size in the donor animals and the size of injury in the recipient animals [49]. Interestingly, MI generates cytotoxic T cells that can kill syngeneic cardiomyocytes in a MHC dependent manner [50].

The induction of MI in the experimental animals showed that the levels of IL-17A and IL-6, which can be produced by Th17 cells, were elevated in the infarcted zone compared to the non-infarcted zone [51] and the implication of γδT cells in the local production of IL-17A [52]. The importance of γδT cells, IL-17A and IL-23 genes in the pathogenesis of MI was demonstrated by using knockout mice when the deletion of any of above mentioned parameters improved animal survival and cardiac function with the reduction of the infarct size [52]. Furthermore, Hofmann and colleagues reported that MI induces the increase in the number of CD3+CD4+ T cells in the myocardium with up-regulation of IFN-γ expression, one of the main pro-inflammatory cytokines produced by Th1 cells, and stimulates proliferation of both conventional CD4+Foxp3− T cells and regulatory CD4+Foxp3+ T cells in the heart-draining lymph nodes. The generation of the adaptive immune response and regulatory T cells plays an important role in the resolution of inflammation since MI in CD4 knockout mice demonstrated an increase in the number of granulocytes and monocytes/macrophages with pro-inflammatory properties in the infarct zone and collagen formation impairment compared to the wild type mice with MI [53]. In addition, it has been shown that the impairment in the recruitment of CD4+Foxp3+ regulatory T cells to the site of tissue injury, which is mediated via CCR5/MIP, causes an increase in the expression of pro-inflammatory cytokines TNF-α, IL-1β and IL-6, and elevates the expression as well as activity of MMP which results in an adverse effect on heart tissue remodeling [54]. The clinical data demonstrated that there is a shift towards the Th1 immune response in patients with acute MI [55], with increased levels of Th1 cells in the blood and IFN-γ in the plasma as well as decreased levels of CD4+CD25+Foxp3+ regulatory T cells in the blood and TGF-β in the plasma [56]. Moreover, the cells of the immune system contribute to scar tissue formation by producing MMP and paracrine factors and by stimulating the migration of fibroblasts [57]. These findings demonstrate that in addition to the innate immune system, the adaptive immune system also plays a major role in tissue damage, clearance of cell debris, and left ventricular remodeling following MI (Figure 1).

Thus, initiation, development and resolution of inflammation in the heart following MI represent a very complex and dynamic process. Consequently, it is crucial to define the balance between detrimental and beneficial effects resulting from the innate and adaptive immune responses in injured myocardium, presumably through paracrine cross-talk and/or cellular interactions between immune cells and various cell populations including cardiac myocytes, endothelial cells, cardiac fibroblasts, and resident/circulating stem cells.

## 3. Cellular Antioxidant Level Represents a Major Determinant in the Cardiac Regenerative Capacity of Stem Cells

The microenvironment after ischemic injury in the cardiac milieu is deleterious to local cells due to oxidative and inflammatory stress, excessive fibrosis, and inadequate angiogenesis [58]. This harsh microenvironment has been suggested as a principal reason for a universally low survival rate of implanted stem cells [59]. Cell survival is an integral and key component of cell-mediated tissue recovery, which can be the result of a reduction in the death of native cells, an increased persistence of donor cells, or a combination of the two [60].

Numerous attempts to repair the infarcted heart using exogenous stem cells have been made; however, very few of the transplanted donor cells actually survive or engraft long-term, a formidable obstacle that needs to be addressed [61]. Alternatively, muscle derived stem cells (MDSCs), a CD34+/Sca1+ stem cell population that has been extensively investigated for tissue repair and regeneration, restored the heart function and tissue structures (increased angiogenesis, reduced ventricular remodeling, and cardiomyocyte differentiation) after ischemic injury more effectively than conventional CD56+ myoblasts [62,63]. This is attributed to, at least in part, the greater number of MDSCs that survived after 12 weeks post-transplantation as well as the robust paracrine function of the MDSCs secreting a number of angiogenic/trophic factors [62]. Nevertheless, it is not clear how the MDSCs survived the unfavorable environment following the ischemic cardiac insult more efficiently than the CD56+ myoblasts.

In an attempt to elucidate the mechanism(s) behind MDSC-mediated heart repair, various cellular functionalities were examined, including apoptosis under oxidative and inflammatory stress. It was found that under conditions of oxidative stress, there were less apoptotic MDSCs than CD56+ myoblasts [62]. This indicates that MDSCs are more resistant to oxidative stress-induced apoptosis than myoblasts. Inflammatory stress-induced cell death was also examined following TNF-α stimulation. The results showed significantly more cell death in the myoblast population compared to the MDSC population, highlighting the unique survival advantage of MDSCs over myoblasts under stressful conditions [64]. Additional analyses revealed that MDSCs exhibit increased levels of the antioxidant glutathione (GSH) and super-oxide dismutase (SOD) as well as decreased levels of ROS after exposure to H_2_O_2_ [64].

To further assess the role of antioxidant capacity after cell transplantation, MDSCs were treated with diethyl maleate (DEM), a thiol-depleting agent that decreases GSH levels. The DEM treatment resulted in a decreased ratio of MDSC engraftment in skeletal muscles, to a similar level seen in myoblast transplantation, indicating that both *in vitro* and *in vivo*, antioxidant levels are critical to the survival and transplantation efficiency of MDSCs [64]. On the other hand, to investigate whether the protection by antioxidant can be augmented, antioxidant levels were increased by treating the MDSCs with the glutathione precursor N-acetylcysteine (NAC). It was shown that NAC treatment of MDSCs increased their survival under oxidative and inflammatory stress *in vitro* compared to untreated MDSCs while, conversely, treatment with DEM decreased their survival [65].

Furthermore, MDSCs pre-treated with NAC 24 hours prior to the intramyocardial transplantation into the ischemic heart notably increased cardiac contractility, compared to untreated MDSCs. There were also improvements in angiogenesis and a reduction of fibrosis. These results indicate that cell survival is indeed an important aspect of cellular therapy for cardiac repair. Methods increasing survival of transplanted cells, as well as native tissue/cells, may substantially aid in the repair process. When the cells are pre-treated with an antioxidant, their capacity to neutralize oxidative species will thus increase, which explains their augmented survival. Other studies have also indicated that cell survival is a key to cardiac cell therapy. Though mesenchymal stem cells have been shown to promote functional and histological improvements after myocardial infarction, when the cells were transduced with the survival factor Akt, this improvement was significantly increased [66]. On another note, VEGF, a potent angiogenic factor, has also been shown to exert a protective effect on the surrounding cardiomyocytes [67]. Consequently, the cell survival and paracrine function of MDSCs appear to be two independent yet inter-related areas where further improvements can be made to further improve cardiac cell therapy.

Similarly, when preconditioned with Carvedilol, which is a nonselective β-blocker with independent antioxidant properties for scavenging superoxide anions and hydroxyl radicals, treated MSCs exhibited significant protection against H_2_O_2_-induced oxidative stress and cell death, compared with untreated cells [68]. Furthermore, transplantation of MSCs with adjuvant treatment of Carvedilol following MI resulted in significant improvement in cardiac function, decreased fibrosis, and reduced cellular apoptosis when compared with the MSC-only group. Additionally, the paracrine mechanism by which MSCs protect cardiomyocyte following ischemia/reperfusion (I/R) injury was further demonstrated by Desantiago *et al*. using an *in vitro* model of I/R [69]. Reperfusion of mouse cardiomyocytes with MSC-conditioned tyrode (MSC-ConT) led to a decreased number of attenuated cardiomyocyte shortening that resulted from depolarization of mitochondrial membrane potential and subsequent increase of diastolic Ca^2+^ as well as prolonged cardiomyocyte survival. The antioxidant capacity of MSC-ConT can be tied, at least partly, to the presence of extracellular superoxide dismutase (SOD3) in the solution, reducing reperfusion-induced ROS production.

## 4. Mesenchymal Stem Cells as Immunomodulators in Cardiac Repair

Mesenchymal stem cells (MSCs), which were initially identified in the bone marrow but can be found and isolated from umbilical cord, placenta, adipose, muscle and other tissues, have attracted the attention of scientists and clinicians as a good source for stem cell-mediated therapy for the following three main reasons. First, MSCs can differentiate into a number of different cell types including cardiac myocytes, endothelial cells, osteoblasts, chondrocytes and adipocytes [70–74]. Second, MSCs are immunoprivileged because of high expression of immunomodulatory MHC-Ib and low or no expression of immunogenic MHC-Ia, MHC-II and co-stimulatory molecules [75,76]. In addition, MSCs possess strong immunosuppressive properties that are mediated via a cell contact-dependent mechanism and production of soluble factors such as prostaglandin E2, nitric oxide, hepatocyte growth factor, TGF-β1, IL-10, indoleamine 2,3-dioxygenase and heme oxygenase-1 [77–81]. However, MSCs become immunogenic after differentiation because of the down-regulation of MHC-Ib and up-regulation of MHC-Ia and MHC-II [76]. Third, MSCs have the ability to migrate to the site of injury and repair the tissue [82,83]. This section of the review is dedicated to the immunomodulatory properties of MSCs in cardiac repair, particularly following acute MI, although their mechanisms of action are complex and still not fully understood. The immunomodulatory effect of MSCs is mainly mediated by paracrine factors because only a minority of injected stem cells is present in the heart tissue [84].

Human MSCs prolong the survival of neutrophils that is mediated by IL-6 with subsequent stimulation of STAT-3 [85,86] and inhibit ROS production, particularly hydrogen peroxide, by stimulated neutrophils that is mediated by IL-6 without affecting their phagocytic and chemotactic activities [86]. A direct myocardial injection of cells that contain human mesenchymal and hematopoietic progenitor cells significantly reduced the number of neutrophils at an earlier time point compared to the control group in a rat experimental model of MI [87]. Moreover, the reduction in the number of recruited neutrophils is probably mediated by a decrease in the levels of pro-inflammatory cytokines such as IFN-γ, TNF-α and IL-1 in the group of animals injected with MSCs.

The systemic administration of human MSCs in the experimental model of acute MI causes a reduction in the total number of monocytes/macrophages in the cardiac tissue. However, the number of CD206+ and F4/80+ cells increased in the group of animals that received MSCs. The increase in the number of alternatively activated/anti-inflammatory macrophages may be mediated by an increase of IL-10 expression and a decrease in the expression of IL-1β and IL-6 at the site of cardiac injury [88]. The *in vitro* data are consistent with the data obtained on experimental animals and demonstrated that human MSCs decrease the number of classically activated mouse bone marrow derived Ly6C+ macrophages and increase the number of alternatively activated CD206+ macrophages, the latter effect is mediated by IL-10 [88]. In addition, the *in vitro* analyses using MSCs and macrophages from the same species confirmed the data obtained on experimental animals. Indeed, mouse MSCs increased the production of IL-10 and IL12p40 while the production of IFN-γ, TNF-α, IL-6 and IL12p70 by activated peritoneal macrophages decreased. The modulatory effect of MSCs was dependent on cell-cell contact and soluble factors, which was partially mediated by prostaglandin E2. Furthermore, mouse MSCs polarized macrophages towards the alternatively activated phenotype with increased phagocytic activity of apoptotic cells and caused the impairment of antigen-presenting capacity [89].

Similar results were reported by other groups using human MSCs and human macrophages. The incubation of human MSCs with human macrophages polarized macrophages toward the alternatively activated/anti-inflammatory phenotype with up-regulation of CD206, CD163 and CD16 as well as the increased secretion of IL-4, IL-10, IL-13 and VEGF, while the levels of IFN-γ, TNF-α, IL-1α, IL-12, IL-17 and IL-23 were decreased [90]. The polarization effect of MSCs on macrophages in these experiments was mediated by IL-6. Kim and colleagues demonstrated that the polarization of monocyte-derived macrophages towards the CD206+ phenotype under the influence of MSCs is mediated by both direct cell-cell contacts and soluble factors [91]. The incubation of human MSCs with stimulated macrophages further increased the intra-cellular levels of IL-10 and IL-6 compared to the control group. In addition, IFN-γ and TNF-α stimulated human MSCs induced the polarization of CD14+ monocytes towards the CD206+ phenotype with significant IL-10 up-regulation, and blocking indoleamine 2,3-dioxygenase partially reduced IL-10 production [92].

There are a number of published papers on the influence of MSCs on adaptive immunity, particularly on T cells, following MI. Indeed, the number of CD3+ and CD4+ T cells decreased at 24 and 72 h after direct myocardial injection in animals that received a population of cells containing mesenchymal and hematopoietic progenitor cells [87]. Similar results were obtained by Burchfield and colleagues who reported that intra-myocardial injection of mouse bone marrow derived mononuclear cells (BM-MNCs) into the mice with MI significantly reduced CD3+ T cell recruitment, which is mediated by IL-10 produced by BM-MNCs [93]. The *in vitro* analysis showed that IFN-γ and TNF-α treated human MSCs suppress the proliferation of nonspecifically stimulated human CD3+ T cells that is directly mediated by indoleamine 2,3-dioxygenase, which catalyzes tryptophan degradation, an important amino acid required for T cell proliferation, and indirectly via the generation of alternatively activated macrophages, which produce IL-10 [92]. In addition to the immunosuppressive ability of MSCs on the T cell population, MSCs contribute to the generation of regulatory T cells. Human MSCs stimulate the generation of CD4+CD25+Foxp3+ regulatory T cells in allogeneic culture of purified human CD4+ T cells, which is mediated through a contact-dependent mechanism and the production of soluble factors such as PGE2 and TGF-β, although the presence of soluble factors alone is not sufficient for the generation of regulatory T cells [94]. Similarly, human MSCs contribute to regulatory T cell generation, which is mediated via the Notch1 signaling pathway [95], and maintain their suppressive activity in a mixed lymphocyte reaction [96]. In the experimental model of acute MI, the adoptive transfer of CD4+CD25+ regulatory T cells improved left ventricular contraction and cardiac function, which may be mediated by the local reduction of IFN-γ levels [97], and by the decrease in the recruitment of neutrophils, macrophages and lymphocytes as well as by the down-regulation of TNF-α and IL-1β expression and elevation of IL-10 at the site of injury [98]. Moreover, CD4+CD25+ regulatory T cells suppress cytotoxic T lymphocyte-mediated lysis of cardiac myocytes and inhibit apoptosis of cardiomyocytes via a contact-dependent mechanism and IL-10 production [98].

In addition to various growth factors and cytokines produced by MSCs, exosomes recently attracted the attention of researchers. Exosomes are small extracellular vesicles that are secreted by a number of cell types and contain RNAs and proteins, which can affect various biological functions. It has been demonstrated that exosomes derived from MSCs reduce infarct size, decrease oxidative stress and cell death in an experimental model of myocardial infarction/reperfusion injury [99,100]. Taken together, the reviewed data indicate that MSCs, through cell-cell contact mechanisms and/or production of soluble factors, create an environment that suppresses ROS production by neutrophils, polarizes monocytes/macrophages towards an alternatively activated/anti-inflammatory phenotype, inhibits proliferation and generation of effector T cells as well as contributes to the increase in the number of regulatory T cells, and as a result, prevents adverse left ventricular remodeling, promotes angiogenesis and improves cardiac function.

## 5. Human Blood Vessel-Derived Stem Cells for Cardiac Repair and Regeneration

Blood vessels throughout the human body are typically composed of three structural layers: intima, media, and adventitia [101]. At the microvascular level, the structure of the vascular wall is reduced to only endothelial cells (ECs) and surrounding perivascular stromal/mural cells, *i.e*., pericytes [102]. It has been suggested that pericytes (PCs) as well as other vascular cell population(s) represent an origin of adult multipotent stem/progenitor cells [103–106]. Recently, our group and other laboratories have successfully identified several subpopulations of multi-lineage precursor cells within the human vasculature, including myogenic endothelial cells (MECs), PCs, and adventitial cells (ACs) [106–108]. Collectively we named these three precursor subpopulations, “human blood-vessel-derived stem cells (hBVSCs)”. These subsets of hBVSCs can be purified from blood vessels using fluorescence-activated cell sorting (FACS), based on their unique profiles of cell surface markers, and share striking similarities to typical mesenchymal stem/stromal cells in culture [105]. Specifically within the human skeletal muscle, MECs and PCs can be identified and isolated from the microvasculature [105–107]. Purified MECs and PCs have been shown to repair/regenerate injured and/or genetically defective tissues more efficiently than vascular ECs and/or conventional CD56+ myoblasts [106,107]. In this section, we summarize the recent progress in the translational applications of MECs and PCs in cardiovascular regenerative medicine.

MECs, a putative developmental intermediate between ECs and myogenic cells (MCs), have been proposed as the human counterpart of murine MDSCs due to their similar anatomical localization and scarcity *in situ* as well as versatile regenerative functionality [109]. MECs not only express MC markers such as CD56 and Pax7, but also express EC markers including CD34, CD144 (VE-cadherin), von Willebrand factor (vWF), and Ulex europaeus agglutinin-1 (UEA-1) [107,110]. Though present at a very low frequency *in situ*, MECs (CD34+/CD56+/CD144+/CD45−) can be purified by FACS to homogeneity. MECs can be expanded efficiently in long-term culture, at the clonal level, and exhibit myogenic, adipogenic, osteogenic, and chondrogenic developmental potentials *in vitro* and *in vivo* [107,109,110]. MECs have also been shown to participate in angiogenesis/vasculogenesis *in vitro* and *in vivo* [109]. The regenerative capacity of MECs in the cardiac milieu has been demonstrated by Okada and colleagues in an ischemic injury model [63]. When transplanted intramyocardially into the ischemic hearts of immunodeficient mice, MECs restored cardiac contractile function more effectively than conventional CD56+ MCs and ECs as is observed with murine MDSCs [62]. Histological analyses showed a significant reduction in cardiac fibrosis and cellular apoptosis as well as augmented angiogenesis and cell proliferation in MEC-injected hearts. These beneficial effects have been largely attributed to the paracrine function of MECs, especially the elevated secretion of VEGF under hypoxia. Nevertheless, transplanted MECs generated robust engraftments and a small number of donor cell-derived cardiomyocytes within the ischemic myocardium, indicating the role of cell survival and differentiation in MEC-mediated cardiac repair [63].

PCs are microvascular mural cells commonly known for their vascular regulatory and supportive functions [111–113], and PC-EC interactions modulate EC proliferation and vascular remodeling [114–116]. Nevertheless, the lack of homogeneous PCs in the past hindered further investigations to explore their regenerative potential. Using similar immunohistochemical and flow cytometry strategies, our group identified and purified PCs from multiple human tissues based on the expression of CD146 (Mel-CAM), NG2 (chondroitin sulphate), platelet-derived growth factor receptor-beta (PDGFRβ), alkaline phosphatase (ALP), with the absence of myogenic (CD56), hematopoietic (CD45), and endothelial (CD31, CD34, and CD144) cell surface markers [106]. Interestingly, only PCs surrounding microvessels (arterioles and venules), but not those around most capillaries, expressed alpha-smooth muscle actin (a-SMA). PCs, natively and in culture, displayed typical MSC differentiation capacities and expressed classic MSC markers: CD44, CD73, CD90 and CD105, indicating their developmental role as precursors of MSCs [106]. The potential applications of FACS-purified PCs (CD146+/CD34−/CD45−/CD56−) in cardiovascular diseases have recently been investigated [117,118]. When transplanted into ischemic hearts of immunodeficient mice, PCs derived from human skeletal muscle were shown to notably improve cardiac contractility and attenuate left ventricular dilatation, superior to CD56+ MCs, for up to 8 weeks [117]. Histological analyses revealed considerable structural recovery in PC-treated hearts, including increased host angiogenesis and substantial reduction of chronic inflammation and myocardial fibrosis at the infarct site. The beneficial effects of PC treatment were attributed to the robust paracrine function of PCs and, to a lesser extent, their direct cellular involvement including perivascular homing, direct interactions with host cells, and differentiation into cardiac cell types [117]. Moreover, PCs were seeded onto small-diameter, bi-layered elastomeric scaffolds to investigate their vascular reparative capacity [118]. After implantation for 8 weeks in Lewis rats, PC-seeded aortic interposition grafts showed a significantly higher rate of vessel patency than unseeded controls and exhibited favorable tissue remodeling including the presence of multiple layers of smooth muscle cells, elastin deposition, and endothelialization in the lumen, suggesting the potential of PCs in vascular tissue engineering.

Apart from MECs and PCs, another distinct subset of hBVSCs, *i.e*., ACs, which resides in the adventitia of human vasculature, has recently been described as a putative pericyte progenitor population [108,119,120]. Campagnolo and colleagues and Katare and colleagues respectively reported that ACs (CD34+/31−) derived from adventitial vasa vasorum of human saphenous vein exert significant therapeutic efficacy in murine ischemic limbs and infarcted hearts, owing primarily to their robust angiogenic/vasculogenic and angiocrine capacities [119,120]. On the other hand, applying the same pre-plating technique used for murine MDSC isolation, our group recently reported the isolation of human slowly adhering cells (hSACs) and explored their therapeutic effect in the ischemic mouse heart [121]. hSACs not only exhibited significantly greater survival under oxidative and inflammatory stress than their rapidly adhering counterparts, but also notably promoted functional and structural recovery of ischemic hearts [121]. Altogether these data indicate robust therapeutic capacities of hBVSC subsets and mMDSC counterparts in cardiovascular regenerative medicine.

## 6. The Role of Human Pericytes in Antioxidation and Immunomodulation

PCs, also known as mural or Rouget cells, are located around endothelial cells in the blood microvessels, incompletely surrounding them, and play a major role in promoting the survival, adhesion and mechanical stabilization of endothelial cells. Moreover, PCs are essential in tissue repair and fibrosis, the development and maintenance of the blood brain barrier, diabetic retinopathy and cancer development [102,122]. In this section, we will review the role of human PCs in antioxidation and immunomodulation.

Using purified MECs and PCs from the same donor, we are currently investigating the antioxidative capacities of these two hBVSC subpopulations under simulated oxidative stress conditions induced by hydrogen peroxide (H_2_O_2_). Our preliminary results showed that PCs proliferated at a higher rate than MECs when stimulated with H_2_O_2_ (100 and 250μM) while more MECs survived in a higher concentration of H_2_O_2_ (400 μM) than the PCs, implicating differential cellular antioxidation in MECs and PCs (Chen and Saparov, unpublished results) [64]. Regarding the published data on the role of PCs in immunomodulations, Stark and colleagues reported [123] that TNF-α and LPS stimulated human placental PCs up-regulated the expression of TLR2, TLR4, TNF receptor and NLRP3 (NLR family, pyrin domain containing 3). The stimulation of PCs by DAMPs as well as TNF-α and LPS also elevated the expression of ICAM-1 on the surface of PCs that is used to bind neutrophils and monocytes via β2-integrins. The analysis of cytokine and chemokine mRNA expression by human placental PCs showed that they constitutively expressed IL-6, macrophage migration inhibitory factor (MIF) and the following chemokines: CXCL1, CXCL8 and CCL2. The stimulation of PCs by DAMPs, as well as with TNF-α and LPS, up-regulated the expression of all cytokines and chemokines;although the levels of up-regulation depended on the stimulus. Moreover, the human placental PCs constitutively express NF-κB and IL-1β, and stimulation with TNF-α increased their expression. The analysis of protein production demonstrated that un-stimulated PCs produced detectable levels of MIF, CXCL1, CXCL8 and CCL2. Both TNF-α and LPS increased MIF production as early as 6 h after stimulation by LPS. However, lysate of necrotic cells increased MIF production 1 h after stimulation, which is indicative of MIF pre-existence within the PCs. Furthermore, the secretion of chemokines was also elevated after stimulation. The combination of secreted MIF and ICAM-1 expressed on the surface of PCs are crucial for their interaction with neutrophils and monocytes via integrins expressed on the surface of these cells. The analysis of factors, which are secreted by human placental PCs and are necessary for the *in vitro* migration of neutrophils and monocytes, revealed that MIF and CXCL8 are required for neutrophil migration, while MIF in combination with CCL2 are implicated in monocyte migration. In addition, TNF-α stimulated PCs activated both neutrophils and monocytes to up-regulate mRNA expression of TLR, MMP and integrins as well as increased the surface expression of CD69 on monocytes, CD11b on neutrophils and extended neutrophil survival [123,124]. These experiments indicate that PCs express pattern recognition receptors to DAMPs and pathogen-associated molecular patterns (PAMPs) and their activation causes the secretion of cytokines and chemokines, some of which are capable of activating neutrophils and monocytes, and influencing their migration which demonstrates the important role of PCs in innate immunity.

Maier and Pober demonstrated [125] that human placental PCs express MHC class I, ICAM-1, PD-L1, PD-L2 without MHC class II and co-stimulatory molecules (CD80 and CD86) expression. The stimulation with IFN-γ in addition, to further increase the expression of MHC-I, ICAM-1, PD-L1 and PD-L2, also up-regulated the expression of MHC-II. Similar results were reported by Verbeek and colleagues using human brain PCs, that in addition to MHC-I and ICAM-1, un-stimulated human brain PCs also express VCAM-1, and stimulation with TNF-α further up-regulated the expression of all three markers. The authors also demonstrated that VLA-4/VCAM-1 is important for T cell interaction with human brain PCs [126]. The experiments with co-culturing placental PCs with allogeneic CD4+ T cells showed that IFN-γ pre-stimulated PCs activated CD4+ T cells to up-regulate the activation markers, CD25 and CD69, to produce very low but detectable levels of IFN-γ and IL-2 without entering into the cell cycle, compared to the un-stimulated PCs. As a result, it caused CD4+ T cells to enter into an anergic state with up-regulation of genes associated with human T cell anergy. However, IFN-γ pre-stimulated PCs are capable of inducing allogeneic CD4+ T cell proliferation if these cells are pre-activated with autologous to PCs cells, which indicate that PCs can activate the effector/memory cells. In addition, both IFN-γ stimulated and un-stimulated placental PCs suppress the CD4+ T cell response to allogeneic cells in a transwell system that is partially inhibited by TGF-β and IL-10 [125,127]. Tu and colleagues also reported that human retinal PCs inhibit the proliferation of activated CD4+ T cells [128]. Our preliminary data showed that un-stimulated human PCs, which were isolated from human skeletal muscle based on the expression of the reported phenotype CD146+/CD34−/CD45−/CD56−, secrete MCP-1 [129]. Both oxidative stress and inflammatory stimulus with LPS further increased MCP-1 production by human PCs while secretion of TGF-β by un-stimulated PCs slightly increased after exposure to oxidative stress *in vitro*, implicating the role of microenvironmental stimulation on the immunomodulatory function of PCs (Chen and Saparov, unpublished results). Overall, the published data demonstrate that human PCs can influence both innate and adaptive immune responses via a cell-cell contact mechanism and by producing soluble factors such as IL-1, IL-6, MIF as well as several chemokines, and can be a good source for stem cell-mediated therapy.

## 7. Conclusions

The lack of oxygen and nutrients following MI triggers a complex and dynamic process that involves the resident cells at the site of infarction, as well as the recruited cells from the circulation and other organs, generating inflammation and oxidative stress and further causing cardiac tissue damage, repair and heart remodeling. Both innate and adaptive immune systems play pivotal roles in this process by producing pro-inflammatory cytokines, reactive oxygen species, proteolytic enzymes as well as chemokines. In addition to damaging cardiac tissue, the cells of the immune system actively clean the site of infarction, resolve inflammation, promote angiogenesis and participate in heart regeneration. Stem cell therapy diminishes the detrimental effect caused by the immune system and contributes to resolving inflammation, as well as improving cardiac function. Understanding the crosstalk between cellular parties involved in this process will facilitate the enhancement of therapeutic approaches in improving cardiac regeneration, potentially eliminating heart failure and reducing mortality following MI.

## References

[1] Alwan A. (2011). Global Status Report on Noncommunicable Diseases 2010.

[2] Mathers C.D., Loncar D. (2006). Projections of global mortality and burden of disease from 2002 to 2030. PLoS Med.

[3] Arslan F., de Kleijn D.P., Pasterkamp G. (2011). Innate immune signaling in cardiac ischemia. Nat. Rev. Cardiol.

[4] Coggins M., Rosenzweig A. (2012). The fire within: Cardiac inflammatory signaling in health and disease. Circ. Res.

[5] Frangogiannis N.G. (2012). Regulation of the inflammatory response in cardiac repair. Circ. Res.

[6] Swirski F.K., Nahrendorf M. (2013). Leukocyte behavior in atherosclerosis, myocardial infarction, and heart failure. Science.

[7] Ptaszek L.M., Mansour M., Ruskin J.N., Chien K.R. (2012). Towards regenerative therapy for cardiac disease. Lancet.

[8] Choudry F.A., Mathur A. (2011). Stem cell therapy in cardiology. Regener. Med.

[9] Anversa P., Kajstura J., Rota M., Leri A. (2013). Regenerating new heart with stem cells. J. Clin. Invest.

[10] Matzinger P. (2002). The danger model: A renewed sense of self. Sci. Signal.

[11] Sha Y., Zmijewski J., Xu Z., Abraham E. (2008). HMGB1 develops enhanced proinflammatory activity by binding to cytokines. J. Immunol.

[12] Yang H., Hreggvidsdottir H.S., Palmblad K., Wang H., Ochani M., Li J., Lu B., Chavan S., Rosas-Ballina M., Al-Abed Y. (2010). A critical cysteine is required for HMGB1 binding to Toll-like receptor 4 and activation of macrophage cytokine release. Proc. Natl. Acad. Sci. USA.

[13] Tsung A., Sahai R., Tanaka H., Nakao A., Fink M.P., Lotze M.T., Yang H., Li J., Tracey K.J., Geller D.A. (2005). The nuclear factor HMGB1 mediates hepatic injury after murine liver ischemia-reperfusion. J. Exp. Med.

[14] Schiraldi M., Raucci A., Muñoz L.M., Livoti E., Celona B., Venereau E., Apuzzo T., de Marchis F., Pedotti M., Bachi A. (2012). HMGB1 promotes recruitment of inflammatory cells to damaged tissues by forming a complex with CXCL12 and signaling via CXCR4. J. Exp. Med.

[15] Kim S., Stice J.P., Chen L., Jung J.S., Gupta S., Wang Y., Baumgarten G., Trial J., Knowlton A.A. (2009). Extracellular heat shock protein 60, cardiac myocytes, and apoptosis. Circ. Res.

[16] Li Y., Si R., Feng Y., Chen H.H., Zou L., Wang E., Zhang M., Warren H.S., Sosnovik D.E., Chao W. (2011). Myocardial ischemia activates an injurious innate immune signaling via cardiac heat shock protein 60 and Toll-like receptor 4. J. Biol. Chem.

[17] Taylor K.R., Yamasaki K., Radek K.A., Nardo A.D., Goodarzi H., Golenbock D., Beutler B., Gallo R.L. (2007). Recognition of hyaluronan released in sterile injury involves a unique receptor complex dependent on Toll-like receptor 4, CD44, and MD-2. Sci. Signal.

[18] Arslan F., Smeets M.B., Vis P.W.R., Karper J.C., Quax P.H., Bongartz L.G., Peters J.H., Hoefer I.E., Doevendans P.A., Pasterkamp G. (2011). Lack of fibronectin-EDA promotes survival and prevents adverse remodeling and heart function deterioration after myocardial infarctionnovelty and significance. Circ. Res.

[19] Medzhitov R., Preston-Hurlburt P., Janeway C.A. (1997). A human homologue of the drosophila Toll protein signals activation of adaptive immunity. Nature.

[20] Oyama J., Blais C., Liu X., Pu M., Kobzik L., Kelly R.A., Bourcier T. (2004). Reduced myocardial ischemia-reperfusion injury in Toll-like receptor 4-deficient mice. Circulation.

[21] Riad A., Jäger S., Sobirey M., Escher F., Yaulema-Riss A., Westermann D., Karatas A., Heimesaat M.M., Bereswill S., Dragun D. (2008). Toll-like receptor-4 modulates survival by induction of left ventricular remodeling after myocardial infarction in mice. J. Immunol.

[22] Shishido T., Nozaki N., Yamaguchi S., Shibata Y., Nitobe J., Miyamoto T., Takahashi H., Arimoto T., Maeda K., Yamakawa M. (2003). Toll-like receptor-2 modulates ventricular remodeling after myocardial infarction. Circulation.

[23] Akira S., Takeda K. (2004). Toll-like receptor signalling. Nat. Rev. Immunol.

[24] Chen C., Kono H., Golenbock D., Reed G., Akira S., Rock K.L. (2007). Identification of a key pathway required for the sterile inflammatory response triggered by dying cells. Nat. Med.

[25] Diepenhorst G.M., van Gulik T.M., Hack C.E. (2009). Complement-mediated ischemia-reperfusion injury: Lessons learned from animal and clinical studies. Annu. Surg.

[26] Banz Y., Rieben R. (2012). Role of complement and perspectives for intervention in ischemia-reperfusion damage. Annu. Med.

[27] Zhang H., Qin G., Liang G., Li J., Barrington R.A., Liu D. (2007). C5aR-mediated myocardial ischemia/reperfusion injury. Biochem. Biophys. Res. Commun.

[28] Van der Pals J., Koul S., Andersson P., Götberg M., Ubachs J., Kanski M., Arheden H., Olivecrona G., Larsson B., Erlinge D. (2010). Treatment with the C5a receptor antagonist ADC-1004 reduces myocardial infarction in a porcine ischemia-reperfusion model. BMC Cardiovasc. Disord.

[29] Ferrari R., Guardigli G., Mele D., Percoco G., Ceconi C., Curello S. (2004). Oxidative stress during myocardial ischaemia and heart failure. Curr. Pharm. Des.

[30] Elahi M.M., Kong Y.X., Matata B.M. (2009). Oxidative stress as a mediator of cardiovascular disease. Oxid. Med. Cell. Longevity.

[31] Tsutsui H., Kinugawa S., Matsushima S. (2011). Oxidative stress and heart failure. Am. J. Physiol.

[32] Palazzo A.J., Jones S.P., Anderson D.C., Granger D.N., Lefer D.J. (1998). Coronary endothelial P-selectin in pathogenesis of myocardial ischemia-reperfusion injury. Am. J. Physiol.

[33] Ivetic A. (2013). Signals regulating l-selectin-dependent leucocyte adhesion and transmigration. Int. J. Biochem. Cell Biol.

[34] Sellak H., Franzini E., Hakim J., Pasquier C. (1994). Reactive oxygen species rapidly increase endothelial ICAM-1 ability to bind neutrophils without detectable upregulation. Blood.

[35] Briaud S.A., Ding Z., Michael L.H., Entman M.L., Daniel S., Ballantyne C.M. (2001). Leukocyte trafficking and myocardial reperfusion injury in ICAM-1/P-selectin-knockout mice. Am. J. Physiol.

[36] Williams M.R., Azcutia V., Newton G., Alcaide P., Luscinskas F.W. (2011). Emerging mechanisms of neutrophil recruitment across endothelium. Trends Immunol.

[37] Entman M.L., Youker K., Shoji T., Kukielka G., Shappell S.B., Taylor A.A., Smith C. (1992). Neutrophil induced oxidative injury of cardiac myocytes. A compartmented system requiring CD11b/CD18-ICAM-1 adherence. J. Clin. Invest.

[38] Soehnlein O., Weber C., Lindbom L. (2009). Neutrophil granule proteins tune monocytic cell function. Trends Immunol.

[39] Nahrendorf M., Swirski F.K., Aikawa E., Stangenberg L., Wurdinger T., Figueiredo J., Libby P., Weissleder R., Pittet M.J. (2007). The healing myocardium sequentially mobilizes two monocyte subsets with divergent and complementary functions. J. Exp. Med.

[40] Leuschner F., Rauch P.J., Ueno T., Gorbatov R., Marinelli B., Lee W.W., Dutta P., Wei Y., Robbins C., Iwamoto Y. (2012). Rapid monocyte kinetics in acute myocardial infarction are sustained by extramedullary monocytopoiesis. J. Exp. Med.

[41] Gregory C.D., Devitt A. (2004). The macrophage and the apoptotic cell: An innate immune interaction viewed simplistically?. Immunology.

[42] Martinez F.O., Helming L., Gordon S. (2009). Alternative activation of macrophages: An immunologic functional perspective. Annu. Rev. Immunol.

[43] Geissmann F., Manz M.G., Jung S., Sieweke M.H., Merad M., Ley K. (2010). Development of monocytes, macrophages, and dendritic cells. Science.

[44] Anzai A., Anzai T., Nagai S., Maekawa Y., Naito K., Kaneko H., Sugano Y., Takahashi T., Abe H., Mochizuki S. (2012). Regulatory role of dendritic cells in postinfarction healing and left ventricular remodeling clinical perspective. Circulation.

[45] Zhu J., Paul W.E. (2010). Peripheral CD4+ T cell differentiation regulated by networks of cytokines and transcription factors. Immunol. Rev.

[46] Locksley R.M. (2009). Nine lives: Plasticity among T helper cell subsets. J. Exp. Med.

[47] Zhou L., Chong M., Littman D.R. (2009). Plasticity of CD4 T cell lineage differentiation. Immunity.

[48] Zhang N., Bevan M.J. (2011). CD8+ T cells: Foot soldiers of the immune system. Immunity.

[49] Maisel A., Cesario D., Baird S., Rehman J., Haghighi P., Carter S. (1998). Experimental autoimmune myocarditis produced by adoptive transfer of splenocytes after myocardial infarction. Circ. Res.

[50] Varda-Bloom N., Leor J., Ohad D.G., Hasin Y., Amar M., Fixler R., Battler A., Eldar M., Hasin D. (2000). Cytotoxic t lymphocytes are activated following myocardial infarction and can recognize and kill healthy myocytes *in vitro*. J. Mol. Cell. Cardiol.

[51] Ávalos A.M., Apablaza F.A., Quiroz M., Toledo V., Peña J.P., Michea L., Irarrazabal C.E., Carrión F.A., Figueroa F.E. (2012). Il-17a levels increase in the infarcted region of the left ventricle in a rat model of myocardial infarction. Biol. Res.

[52] Yan X., Shichita T., Katsumata Y., Matsuhashi T., Ito H., Ito K., Anzai A., Endo J., Tamura Y., Kimura K. (2012). Deleterious effect of the IL-23/IL-17a axis and γδt cells on left ventricular remodeling after myocardial infarction. J. Am. Heart Assoc.

[53] Hofmann U., Beyersdorf N., Weirather J., Podolskaya A., Bauersachs J., Ertl G., Kerkau T., Frantz S. (2012). Activation of CD4 T lymphocytes improves wound healing and survival after experimental myocardial infarction in mice clinical perspective. Circulation.

[54] Dobaczewski M., Xia Y., Bujak M., Gonzalez-Quesada C., Frangogiannis N.G. (2010). CCR5 signaling suppresses inflammation and reduces adverse remodeling of the infarcted heart, mediating recruitment of regulatory t cells. Am. J. Pathol.

[55] Cheng X., Liao Y., Ge H., Li B., Zhang J., Yuan J., Wang M., Liu Y., Guo Z., Chen J. (2005). TH1/TH2 functional imbalance after acute myocardial infarction: coronary arterial inflammation or myocardial inflammation. J. Clin. Immunol.

[56] Zhao Z., Wu Y., Cheng M., Ji Y., Yang X., Liu P., Jia S., Yuan Z. (2011). Activation of Th17/Th1 and Th1, but not Th17, is associated with the acute cardiac event in patients with acute coronary syndrome. Atherosclerosis.

[57] Van den Borne S.W.M., Diez J., Blankesteijn W.M., Verjans J., Hofstra L., Narula J. (2009). Myocardial remodeling after infarction: The role of myofibroblasts. Nat. Rev. Cardiol.

[58] Hausenloy D.J., Yellon D.M. (2013). Myocardial ischemia-reperfusion injury: A neglected therapeutic target. J. Clin. Invest.

[59] Segers V.F., Lee R.T. (2008). Stem-cell therapy for cardiac disease. Nature.

[60] Haider H.K., Ashraf M. (2010). Preconditioning and stem cell survival. J. Cardiovas. Transl. Res.

[61] Deutsch M., Sturzu A., Wu S.M. (2013). At a crossroad cell therapy for cardiac repair. Circ. Res.

[62] Oshima H., Payne T.R., Urish K.L., Sakai T., Ling Y., Gharaibeh B., Tobita K., Keller B.B., Cummins J.H., Huard J. (2005). Differential myocardial infarct repair with muscle stem cells compared to myoblasts. Mol. Therapy.

[63] Okada M., Payne T.R., Zheng B., Oshima H., Momoi N., Tobita K., Keller B.B., Phillippi J.A., Péault B., Huard J. (2008). Myogenic endothelial cells purified from human skeletal muscle improve cardiac function after transplantation into infarcted myocardium. J. Am. Coll. Cardiol.

[64] Urish K.L., Vella J.B., Okada M., Deasy B.M., Tobita K., Keller B.B., Cao B., Piganelli J.D., Huard J. (2009). Antioxidant levels represent a major determinant in the regenerative capacity of muscle stem cells. Mol. Biol. Cell.

[65] Drowley L., Okada M., Beckman S., Vella J., Keller B., Tobita K., Huard J. (2010). Cellular antioxidant levels influence muscle stem cell therapy. Mol. Therapy.

[66] Mangi A.A., Noiseux N., Kong D., He H., Rezvani M., Ingwall J.S., Dzau V.J. (2003). Mesenchymal stem cells modified with AKT prevent remodeling and restore performance of infarcted hearts. Nat. Med.

[67] Jiang S., Haider H.K., Idris N.M., Salim A., Ashraf M. (2006). Supportive interaction between cell survival signaling and angiocompetent factors enhances donor cell survival and promotes angiomyogenesis for cardiac repair. Circ. Res.

[68] Hassan F., Meduru S., Taguchi K., Kuppusamy M.L., Mostafa M., Kuppusamy P., Khan M. (2012). Carvedilol enhances mesenchymal stem cell therapy for myocardial infarction via inhibition of caspase-3 expression. J. Pharmacol. Exp. Ther.

[69] DeSantiago J., Bare D.J., Banach K. (2013). Ischemia-reperfusion injury protection by mesenchymal stem cell derived antioxidant capacity. Stem Cells Dev..

[70] Pittenger M.F., Mackay A.M., Beck S.C., Jaiswal R.K., Douglas R., Mosca J.D., Moorman M.A., Simonetti D.W., Craig S., Marshak D.R. (1999). Multilineage potential of adult human mesenchymal stem cells. Science.

[71] Toma C., Pittenger M.F., Cahill K.S., Byrne B.J., Kessler P.D. (2002). Human mesenchymal stem cells differentiate to a cardiomyocyte phenotype in the adult *Murine* heart. Circulation.

[72] Jiang Y., Jahagirdar B.N., Reinhardt R.L., Schwartz R.E., Keene C.D., Ortiz-Gonzalez X.R., Reyes M., Lenvik T., Lund T., Blackstad M. (2002). Pluripotency of mesenchymal stem cells derived from adult marrow. Nature.

[73] Amado L.C., Saliaris A.P., Schuleri K.H., John M.S., Xie J., Cattaneo S., Durand D.J., Fitton T., Kuang J.Q., Stewart G. (2005). Cardiac repair with intramyocardial injection of allogeneic mesenchymal stem cells after myocardial infarction. Proc. Natl. Acad. Sci. USA.

[74] Miyahara Y., Nagaya N., Kataoka M., Yanagawa B., Tanaka K., Hao H., Ishino K., Ishida H., Shimizu T., Kangawa K. (2006). Monolayered mesenchymal stem cells repair scarred myocardium after myocardial infarction. Nat. Med.

[75] Gebler A., Zabel O., Seliger B. (2012). The immunomodulatory capacity of mesenchymal stem cells. Trends Mol. Med.

[76] Huang X., Sun Z., Miyagi Y., Kinkaid H.M., Zhang L., Weisel R.D., Li R. (2010). Differentiation of allogeneic mesenchymal stem cells induces immunogenicity and limits their long-term benefits for myocardial repairclinical perspective. Circulation.

[77] Duffy M.M., Pindjakova J., Hanley S.A., McCarthy C., Weidhofer G.A., Sweeney E.M., English K., Shaw G., Murphy J.M., Barry F.P. (2011). Mesenchymal stem cell inhibition of T-helper 17 cell-differentiation is triggered by cell-cell contact and mediated by prostaglandin E2 via the ep4 receptor. Eur. J. Immunol.

[78] Shi Y., Hu G., Su J., Li W., Chen Q., Shou P., Xu C., Chen X., Huang Y., Zhu Z. (2010). Mesenchymal stem cells: A new strategy for immunosuppression and tissue repair. Cell Res.

[79] DelaRosa O., Lombardo E., Beraza A., Mancheño-Corvo P., Ramirez C., Menta R., Rico L., Camarillo E., García L., Abad J.L. (2009). Requirement of IFN-γ–mediated indoleamine 2,3-dioxygenase expression in the modulation of lymphocyte proliferation by human adipose-derived stem cells. Tissue Eng, Part A.

[80] Chabannes D., Hill M., Merieau E., Rossignol J., Brion R., Soulillou J.P., Anegon I., Cuturi M.C. (2007). A role for heme oxygenase-1 in the immunosuppressive effect of adult rat and human mesenchymal stem cells. Blood.

[81] Uccelli A., Moretta L., Pistoia V. (2008). Mesenchymal stem cells in health and disease. Nat. Rev. Immunol.

[82] Bernardo M.E., Fibbe W.E. (2012). Safety and efficacy of mesenchymal stromal cell therapy in autoimmune disorders. Annu. N.Y. Acad. Sci.

[83] Williams A.R., Hare J.M. (2011). Mesenchymal stem cells biology, pathophysiology, translational findings, and therapeutic implications for cardiac disease. Circ. Res.

[84] Ranganath S.H., Levy O., Inamdar M.S., Karp J.M. (2012). Harnessing the mesenchymal stem cell secretome for the treatment of cardiovascular disease. Cell.

[85] Cassatella M.A., Mosna F., Micheletti A., Lisi V., Tamassia N., Cont C., Calzetti F., Pelletier M., Pizzolo G., Krampera M. (2011). Toll-like receptor-3-activated human mesenchymal stromal cells significantly prolong the survival and function of neutrophils. Stem Cells.

[86] Raffaghello L., Bianchi G., Bertolotto M., Montecucco F., Dallegri F., Ottonello L., Pistoia V. (2008). Human mesenchymal stem cells inhibit neutrophil apoptosis: A model for neutrophil preservation in the bone marrow niche. Stem Cells.

[87] Henning R.J., Shariff M., Eadula U., Alvarado F., Vasko M., Sanberg P.R., Sanberg C.D., Delostia V. (2008). Human cord blood mononuclear cells decrease cytokines and inflammatory cells in acute myocardial infarction. Stem Cells Dev.

[88] Dayan V., Yannarelli G., Billia F., Filomeno P., Wang X., Davies J.E., Keating A. (2011). Mesenchymal stromal cells mediate a switch to alternatively activated monocytes/macrophages after acute myocardial infarction. Basic Res. Cardiol.

[89] Maggini J., Mirkin G., Bognanni I., Holmberg J., Piazzón I.M., Nepomnaschy I., Costa H., Cañones C., Raiden S., Vermeulen M. (2010). Mouse bone marrow-derived mesenchymal stromal cells turn activated macrophages into a regulatory-like profile. PLoS One.

[90] Adutler-Lieber S., Ben-Mordechai T., Naftali-Shani N., Asher E., Loberman D., Raanani E., Leor J. (2013). Human macrophage regulation via interaction with cardiac adipose tissue-derived mesenchymal stromal cells. J. Cardiovasc. Pharmacol. Ther.

[91] Kim J., Hematti P. (2009). Mesenchymal stem cell-educated macrophages: A novel type of alternatively activated macrophages. Exp. Hematol.

[92] François M., Romieu-Mourez R., Li M., Galipeau J. (2011). Human MSC suppression correlates with cytokine induction of indoleamine 2, 3-dioxygenase and bystander m2 macrophage differentiation. Mol. Ther.

[93] Burchfield J.S., Iwasaki M., Koyanagi M., Urbich C., Rosenthal N., Zeiher A.M., Dimmeler S. (2008). Interleukin-10 from transplanted bone marrow mononuclear cells contributes to cardiac protection after myocardial infarction. Circ. Res.

[94] English K., Ryan J., Tobin L., Murphy M., Barry F., Mahon B. (2009). Cell contact, prostaglandin E2 and transforming growth factor beta 1 play non-redundant roles in human mesenchymal stem cell induction of CD4 CD25 highforkhead box P3 regulatory T cells. Clin. Exp. Immunol.

[95] Del Papa B., Sportoletti P., Cecchini D., Rosati E., Balucani C., Baldoni S., Fettucciari K., Marconi P., Martelli M.F., Falzetti F. (2013). Notch1 modulates mesenchymal stem cells mediated regulatory T-cell induction. Eur. J. Immunol.

[96] Di Ianni M., del Papa B., de Ioanni M., Moretti L., Bonifacio E., Cecchini D., Sportoletti P., Falzetti F., Tabilio A. (2008). Mesenchymal cells recruit and regulate t regulatory cells. Exp. Hematol.

[97] Matsumoto K., Ogawa M., Suzuki J., Hirata Y., Nagai R., Isobe M. (2011). Regulatory T lymphocytes attenuate myocardial infarction-induced ventricular remodeling in mice. Int. Heart J.

[98] Tang T., Yuan J., Zhu Z., Zhang W., Xiao H., Xia N., Yan X., Nie S., Liu J., Zhou S. (2012). Regulatory T cells ameliorate cardiac remodeling after myocardial infarction. Basic Res. Cardiol.

[99] Lai R.C., Arslan F., Lee M.M., Sze N.S.K., Choo A., Chen T.S., Salto-Tellez M., Timmers L., Lee C.N., el Oakley R.M. (2010). Exosome secreted by msc reduces myocardial ischemia/reperfusion injury. Stem Cell Res.

[100] Arslan F., Lai R.C., Smeets M.B., Akeroyd L., Choo A., Aguor E.N., Timmers L., van Rijen H.V., Doevendans P.A., Pasterkamp G. (2013). Mesenchymal stem cell-derived exosomes increase ATP levels, decrease oxidative stress and activate PI3K/Akt pathway to enhance myocardial viability and prevent adverse remodeling after myocardial ischemia/reperfusion injury. Stem Cell Res.

[101] Kumar V., Abbas A.K., Fausto N., Aster J.C. (2009). Robbins & Cotran Pathologic Basis of Disease.

[102] Armulik A., Genové G., Betsholtz C. (2011). Pericytes: Developmental, physiological, and pathological perspectives, problems, and promises. Dev. Cell.

[103] Caplan A.I. (2008). All MSCs are pericytes?. Cell Stem Cell.

[104] Chen C., Montelatici E., Crisan M., Corselli M., Huard J., Lazzari L., Péault B. (2009). Perivascular multi-lineage progenitor cells in human organs: Regenerative units, cytokine sources or both?. Cytokine Growth Factor Rev.

[105] Chen C., Corselli M., Péault B., Huard J. (2012). Human blood-vessel-derived stem cells for tissue repair and regeneration. Biomed. Res. Int..

[106] Crisan M., Yap S., Casteilla L., Chen C., Corselli M., Park T.S., Andriolo G., Sun B., Zheng B., Zhang L. (2008). A perivascular origin for mesenchymal stem cells in multiple human organs. Cell Stem Cell.

[107] Zheng B., Cao B., Crisan M., Sun B., Li G., Logar A., Yap S., Pollett J.B., Drowley L., Cassino T. (2007). Prospective identification of myogenic endothelial cells in human skeletal muscle. Nat. Biotechnol.

[108] Corselli M., Chen C., Sun B., Yap S., Rubin J.P., Péault B. (2012). The tunica adventitia of human arteries and veins as a source of mesenchymal stem cells. Stem Cells Dev.

[109] Zheng B., Li G., Chen W.C., Deasy B.M., Pollett J.B., Sun B., Drowley L., Gharaibeh B., Usas A., Péault B. (2013). Human myogenic endothelial cells exhibit chondrogenic and osteogenic potentials at the clonal level. J. Orthop. Res.

[110] Zheng B., Chen C., Li G., Thompson S.D., Poddar M., Peault B., Huard J. (2012). Isolation of myogenic stem cells from cultures of cryopreserved human skeletal muscle. Cell Transplant.

[111] Rucker H.K., Wynder H.J., Thomas W.E. (2000). Cellular mechanisms of CNS pericytes. Brain Res. Bull.

[112] Hellström M., Gerhardt H., Kalén M., Li X., Eriksson U., Wolburg H., Betsholtz C. (2001). Lack of pericytes leads to endothelial hyperplasia and abnormal vascular morphogenesis. J. Cell Biol.

[113] Von Tell D., Armulik A., Betsholtz C. (2006). Pericytes and vascular stability. Exp. Cell Res.

[114] Armulik A., Abramsson A., Betsholtz C. (2005). Endothelial/pericyte interactions. Circ. Res.

[115] Gaengel K., Genové G., Armulik A., Betsholtz C. (2009). Endothelial-mural cell signaling in vascular development and angiogenesis. Arterioscler. Thromb Vasc. Biol.

[116] Dulmovits B.M., Herman I.M. (2012). Microvascular remodeling and wound healing: A role for pericytes. Int. J. Biochem. Cell Biol.

[117] Chen C., Okada M., Proto J.D., Gao X., Sekiya N., Beckman S.A., Corselli M., Crisan M., Saparov A., Tobita K. (2013). Human pericytes for ischemic heart repair. Stem Cells.

[118] He W., Nieponice A., Soletti L., Hong Y., Gharaibeh B., Crisan M., Usas A., Peault B., Huard J., Wagner W.R. (2010). Pericyte-based human tissue engineered vascular grafts. Biomaterials.

[119] Campagnolo P., Cesselli D., Zen A.A.H., Beltrami A.P., Kränkel N., Katare R., Angelini G., Emanueli C., Madeddu P. (2010). Human adult vena saphena contains perivascular progenitor cells endowed with clonogenic and proangiogenic potential. Circulation.

[120] Katare R., Riu F., Mitchell K., Gubernator M., Campagnolo P., Cui Y., Fortunato O., Avolio E., Cesselli D., Beltrami A.P. (2011). Transplantation of human pericyte progenitor cells improves the repair of infarcted heart through activation of an angiogenic program involving micro-RNA-132 novelty and significance. Circ. Res.

[121] Okada M., Payne T.R., Drowley L., Jankowski R.J., Momoi N., Beckman S., Chen W.C., Keller B.B., Tobita K., Huard J. (2012). Human skeletal muscle cells with a slow adhesion rate after isolation and an enhanced stress resistance improve function of ischemic hearts. Mol. Ther.

[122] Quaegebeur A., Lange C., Carmeliet P. (2011). The neurovascular link in health and disease: Molecular mechanisms and therapeutic implications. Neuron.

[123] Stark K., Eckart A., Haidari S., Tirniceriu A., Lorenz M., von Brühl M., Gärtner F., Khandoga A.G., Legate K.R., Pless R. (2012). Capillary and arteriolar pericytes attract innate leukocytes exiting through venules and “instruct” them with pattern-recognition and motility programs. Nat. Immunol.

[124] Alon R., Nourshargh S. (2012). Learning in motion: Pericytes instruct migrating innate leukocytes. Nat. Immunol.

[125] Maier C.L., Pober J.S. (2011). Human placental pericytes poorly stimulate and actively regulate allogeneic CD4 T Cell responses. Arterioscler. Thromb Vasc. Biol.

[126] Verbeek M.M., Westphal J.R., Ruiter D.J., de Waal R. (1995). T lymphocyte adhesion to human brain pericytes is mediated via very late Antigen-4/vascular cell adhesion Molecule-1 interactions. J. Immunol.

[127] Pober J.S., Tellides G. (2012). Participation of blood vessel cells in human adaptive immune responses. Trends Immunol.

[128] Tu Z., Li Y., Smith D.S., Sheibani N., Huang S., Kern T., Lin F. (2011). Retinal pericytes inhibit activated t cell proliferation. Invest. Ophthalmol. Vis. Sci.

[129] Chen C.-W., Saparov A. (2013).

